# Non Ionising Radiation as a Non Chemical Strategy in Regenerative Medicine: Ca^2+^-ICR *“In Vitro”* Effect on Neuronal Differentiation and Tumorigenicity Modulation in NT2 Cells

**DOI:** 10.1371/journal.pone.0061535

**Published:** 2013-04-09

**Authors:** Mario Ledda, Francesca Megiorni, Deleana Pozzi, Livio Giuliani, Enrico D’Emilia, Sara Piccirillo, Cristiana Mattei, Settimio Grimaldi, Antonella Lisi

**Affiliations:** 1 Institute of Translational Pharmacology, National Research Council, Rome, Italy; 2 Department of Experimental Medicine, University of Rome “Sapienza”, Rome, Italy; 3 Department of Productive Plants and Interaction with the Environment, National Institute for Occupational Safety and Prevention, Rome, Italy; Institute of Clinical Physiology, c/o Toscana Life Sciences Foundation, Italy

## Abstract

In regenerative medicine finding a new method for cell differentiation without pharmacological treatment or gene modification and minimal cell manipulation is a challenging goal. In this work we reported a neuronal induced differentiation and consequent reduction of tumorigenicity in NT2 human pluripotent embryonal carcinoma cells exposed to an extremely low frequency electromagnetic field (ELF-EMF), matching the cyclotron frequency corresponding to the charge/mass ratio of calcium ion (Ca^2+^-ICR). These cells, capable of differentiating into post-mitotic neurons following treatment with Retinoic Acid (RA), were placed in a solenoid and exposed for 5 weeks to Ca^2+^-ICR. The solenoid was installed in a μ-metal shielded room to avoid the effect of the geomagnetic field and obtained totally controlled and reproducible conditions. Contrast microscopy analysis reveled, in the NT2 exposed cells, an important change in shape and morphology with the outgrowth of neuritic-like structures together with a lower proliferation rate and metabolic activity alike those found in the RA treated cells. A significant up-regulation of early and late neuronal differentiation markers and a significant down-regulation of the transforming growth factor-α (TGF-α) and the fibroblast growth factor-4 (FGF-4) were also observed in the exposed cells. The decreased protein expression of the transforming gene Cripto-1 and the reduced capability of the exposed NT2 cells to form colonies in soft agar supported these last results. In conclusion, our findings demonstrate that the Ca^2+^-ICR frequency is able to induce differentiation and reduction of tumorigenicity in NT2 exposed cells suggesting a new potential therapeutic use in regenerative medicine.

## Introduction

Human NTera2/CloneD1 (NT2) are pluripotent embryonal carcinoma cells derived from a metastasis of a human testicular germ cell tumor (TGCT) providing an important tool to study human neurogenesis “*in vitro”* [1]. During differentiation, sequential activation of Ngn1 (neurogenin 1), Mash1 (mammalian homologue of Drosophila achaete-scute gene), NeuroD (neuronal differentiation), Math1 (mammalian homologue of Drosophila atonal gene) and Pax6 (Paired box) converts pluripotent NT2 cells into a pool of different neuronal subtypes of the central nervous system (CNS). In particular, Mash1 leads to the formation of noradrenergic, GABAergic and cholinergic neurons [2] while Ngn1 and NeuroD direct to glutamatergic and sensor neurons [3].

Also differentiated cells, showed the expression of mRNA calcium channels and the cytosolic calcium transients blocked by nifedipine and v-conotoxin GVIA [4], demonstrating the functional expression of N- and L-type calcium channels in them [5]. NT2 cells also have similar properties as embryonic stem (ES) cells, their pluripotency is maintained by the expression of a set of genes, some of which are down-regulated through the induction of differentiation.

Undifferentiated embryonal carcinoma cells also express genes involved in the stimulation, and the inhibition of cell proliferation and in the direct malignant growth promotion, such as Transforming Growth Factor-α (TGF-α) and Fibroblast Growth Factor-4 (FGF-4) respectively, but also retain cellular malignant features through the expression of Teratocarcinoma-Derived Growth Factor-1 (TDGF-1), also known as Cripto-1. [6], [7]. These cells transplanted in nude mice rapidly progressed into lethal tumors [8], [9].

Recent studies investigated and reported that the treatment with retinoic acid (RA) is able to block the NT2 capacity of malignant transformation both *“in vitro”* and *“in vivo”* [10] and to commit cells into post-mitotic neurons, that exhibit an extensive network of axonal and dendrite processes [11], [12], [13], suggesting their potential application in neuronal regenerative medicine [14], [15].

In particular during RA-induced differentiation, the reduction of tumorigenicity occurs simultaneously with the down-regulation of TGF-α, FGF-4 and Cripto-1 [16], [17] and the up-regulation of specific neural basic helix-loop-helix (bHLH) transcription factors [1], [18], [19].

Another important thing in these cells is an altered expression of the aurora kinases family [20], [21], recently observed, associated with malignant cell transformation and genomic instability, [22]. In these studies the aurora Kinase inhibitors were identified and investigated for their capability to reduce *“in vitro”* NT2 growth and tumorigenicity in order to find a new potential application in cancer therapies [22], [23], [24], [25].

In the light of these findings, new therapeutic approaches are being studied for all those cancer cases resistant to conventional chemotherapy treatments with serious clinical problems [26], [27], [28].

In this paper, our knowledge on the biological interaction effects of ELF-EMFs on cell proliferation [29], [30] and lineage-specific commitment in both mouse and human cells and stem cells, [31], [32], [33], [34] has induced us to investigate the possible use of electromagnetic field exposure as a tool for a new differentiation method to be applied in cell therapy.

We have therefore studied the effects of the Ca^2+^-ICR exposure on pluripotent embryonal carcinoma NT2 cells in order to drive their differentiation towards a neuronal phenotype and consequently to reduce the “*in vitro*” tumorigenicity.

## Materials and Methods

### Cell culture

Human NTera2/Clone D1 (NT2) was obtained from the American Type Culture Collection (ATCC® Number: CRL-1973™). The cells were grown in high-glucose Dulbecco's modified Eagle's Medium (DMEM; Euroclone) supplemented with 10% heat-inactivated fetal bovine serum (FBS, Euroclone), 2 mM L-glutamine (Sigma), 1.0 unit/ml penicillin (Sigma), and 1.0 mg/ml streptomycin (Sigma). The human NT2 cell line was cultured at 37°C in a humidified incubator containing 5% CO_2_. In our paper the cells have been seeded on bacterial Petri dishes at a concentration of 4×10^5^/ml to allow the growth and development of cellular floating aggregates (also called spheres). After an overnight incubation, the cells were cultured and treated for 5 weeks in 3 different conditions: they were grown in absence (control) and in presence of Ca^2+^-ICR frequency (exposed, 7 Hz, 2.5 µT) and also treated with retinoic acid (RA, 5 µM Sigma) which was used as positive control. Medium and dishes have been replaced every 3 days. To study cell morphology at the end of treatments, the cellular spheres have been plated into matrigel precoated- Petri dishes, and cultured for an additional week in a medium supplemented with 10 µg/ml cytosine β-D-arabinofuranoside (AraC) [1], [35].

### Exposure system description and characterization

**Figure 1:**
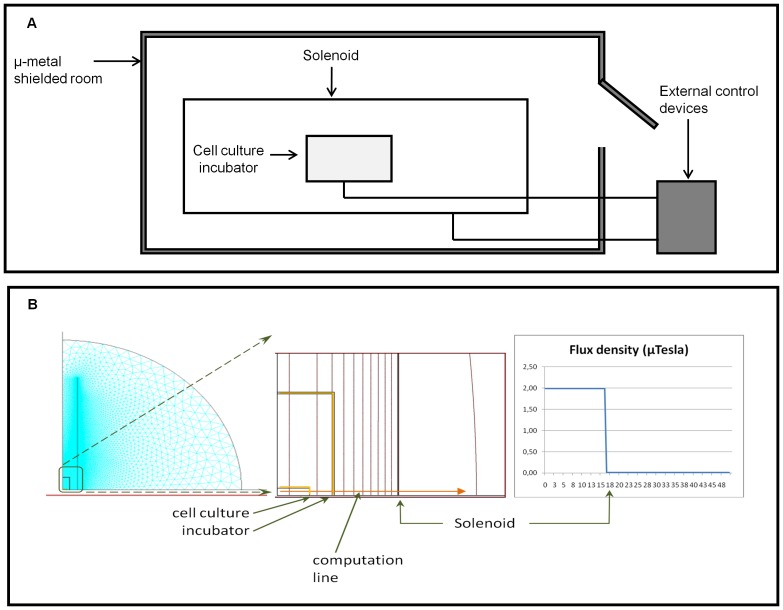
Schematic representation and quantitative description for the Ca^2+^-ICR exposure apparatus for electromagnetic field generation. (A) The Ca^2+^-ICR exposure system illustration. (B) Magnitude and flux lines of the magnetic flux density of the overall exposure system (zoom on the right).

The equipment for electro-magnetic field production (solenoid) is set-up in the μ-metal shielded room located in our laboratories [30], [32]. Briefly the apparatus includes a cellular incubator, made of polymethylmethacrylate (PMMA), a diamagnetic material that have a relative permeability less than 1, where temperature (37± 0.1°C), atmosphere composition (5% CO_2_) and humidity regulation is provided, continuously controlled and recorded by a lab view program (control system). The main body of home produced solenoid is a cylinder in polyvinyl chloride, 5 mm thick, with a diameter of 33 cm and is 3.3 m long. It is made of 3,300 turns of 1 mm diameter copper wire. It is driven by three amplifiers and a signal generator that create static and alternate current for EMF production (Figure 1). A magnetic field with frequencies of 0.01 Hz to 1 KHz is produced by the equipment, and a magnetic flux density which magnitude spans between 10 nT and 15]mT; the maximum output voltage of the voltage supplier which feeds the solenoid is 33 mV RMS. The control experiment was run in the second of the twin diamagnetic incubators, placed outside the shielded room, where the geomagnetic field, measured along the same direction of the one in the shielded room, resulted to be 18 µT, while the positive control cell experiments were run in a normal cell incubator ThermoForma 3111 (Thermo scientific). In both diamagnetic incubators, the atmosphere, humidity and temperature were simultaneously and electronically controlled and recorded.

### Ca^2+^-ICR exposure parameters

The exposure parameters were calculated based on the following Lorentz's equation:



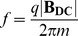
where *q* and *m* are respectively the ion's charge and mass, |**B**_DC_| is the flux density of the applied static MF, and *f* is the frequency of the superimposed EMF [36].

We applied a very low intensity of EMF (in the range of µT) since at resonance condition it is hypothesized that a maximum energy transfer occurs enabling us to see a biological effect. Under these conditions the amount of heating due to the Joule effect was negligible, and all the effects reported after cells exposure are related to the calcium ion cyclotron resonance exposure. In our study, NT2cells were continuously exposed up to 5 weeks to a static MF (10 µT) and an alternating EMF (2.5 µT RMS of intensity) at 7 Hz, matching the cyclotron frequency corresponding to the charge/mass ratio of calcium ion (Ca^2+^-ICR).

The exposure parameters were chosen according to our previous results showing a differentiation effect on human cardiac stem cells and mouse skeletal muscle cells [30], [32]. The experiment with cells that were not exposed (control cells) was carried out in a twin diamagnetic cell incubator where the temperature (37± 0.1°C), atmosphere composition (5% CO_2_) and humidity regulation was remotely controlled by a lab view program such as the one exposed into the solenoid. All experiments have been performed under single-blind conditions, the samples (exposed and control not exposed cells) were numbered and the operator did not know which ones were being used.

### Cell morphology analysis

The NT2cells were seeded and cultured on bacterial Petri dishes as spheres and treated for 5 weeks in 3 different conditions: they were grown in absence (control) and in presence of Ca^2+^-ICR frequency (exposed). The control cells were also treated with retinoic acid which was carried out as positive control. At the end of these treatments the cellular spheres were seeded on precoated matrigel cover glasses and cultured for an additional week in these conditions. The cells were, then washed in PBS, fixed in paraformaldeyde 4% in PBS for 15 min, and tested by phase contrast microscopy to observe cell morphology and to visualize the presence of neuritic structures. Phase contrast analysis were performed using an inverted microscope (Olympus IX51, RT Slider SPOT - Diagnostic instruments) equipped with a 20×, 4×and 60×objective and with a cooled CCD camera (Spot RT Slider, Diagnostic Instruments).

### Cell metabolic activity analysis

The NT2 cells were seeded and cultured on bacterial Petri dishes as spheres in absence (control), or in presence (exposed) of Ca^2+^-ICR frequency for 5 weeks. The control cells were also treated with retinoic acid which was carried out as positive control. Water soluble tetrazolium salt (WST-1) reagent diluted to 1∶10 was added in the medium at 1, 2, 3, 4 and 5 weeks and after an incubation of 2 h in a humidified atmosphere, the supernatants (100 µl) of NT2 cells were put in 96-well plates and analyzed by means of formazan dye (Cell Proliferation Reagent WST-1; Roche Diagnostics). The quantification of NT2 metabolic activity was performed by a colorimetric assay based on oxidation of tetrazolium salts by absorbance measurement at 450 nm with a scanning multiwell spectrophotometer (Biotrack II; Amersham Biosciences).

### Cell proliferation analysis

The NT2 cell proliferation was evaluated by Bromodeoxyuridine incorporation assay (BrdU).

The NT2 cells were seeded and cultured, as spheres, on bacterial Petri dishes and treated for 5 weeks in 3 different conditions: they were grown in absence (control) and in presence of Ca^2+^-ICR frequency (exposed) and also treated with retinoic acid which was used as positive control. Bromodeoxyuridine 10 mM was added in the NT2 medium at 1, 2, 3, 4 and 5 weeks and maintained for 18 hours in culture. Cells were then fixed and incubated for 30 min at room temperature with the anti-BrdU antibody (Cell Proliferation Kit; Roche Diagnostics). After incubation with 2,20-Azino-bis (3-ethylbenzothiazoline-6-sulfonic acid) for 30 min, the absorbance of 100 µl of supernatant was measured in an ELISA reader at 450 nm.

### Real-Time quantitative RT-PCR analysis

Total RNA was extracted after 1, 2, 3, 4 and 5 weeks from control NT2 cells, exposed and RA treated cells, using TRIzol Reagent (Life Technologies), according to the manufacturer's instructions. One microgram of total RNA was used to synthesize first-strand cDNA with random primers, using 100 U of ImProm-II ™ RT-PCR kit (Promega). The quantification of every mRNAs was carried out by real-time quantitative RT-PCR. Experiments were conducted to obtain relative levels of each transcript normalized for the endogenous control GAPDH in every sample. Gene expression was presented using the -2^-ΔΔCt^ method, described by [37] where ΔCt represents (average target Ct – average GAPDH Ct), and ΔΔCt represents (average ΔCt treated sample – average ΔCt untreated sample).

We performed a validation experiment to demonstrate that the amplification efficiency of target genes and reference GAPDH was equal and that the GAPDH mRNA expression was the same among the different treatments. Real-time PCR was performed with Sybr Green I Mastermix (Applied Biosystems) using an ABI PRISM™7000 Sequence Detection System. Each reaction was run in triplicate and contained 1 µl of cDNA template with 250 nM primers in a final reaction volume of 25 µl. The specific primers and annealing temperatures used are shown in Table 1. Cycling parameters were: 50°C for 2 min, 95°C for 10 min to activate DNA polymerase, then 40-45 cycles of 95°C for 15 s and 60°C for 1 min. Melting curves were performed using Dissociation Curves software (Applied Biosystems) to ensure only a single product was amplified. As negative controls, reactions were prepared in which RNA or reverse transcriptase were previously omitted during reverse trascription.

**Table 1:** Primers used for real-time reverse transcriptase polymerase chain reaction.

Target	Sequence	Accession numbers	Annealing temperature
NeuroD	5′-GGAATTCGCCCACGCAGG-3′	NM_002500	60°C
	5′-CCCATCAGCCCACTCTCG-3′		
NR1	5′-CAGATGGCAAGTTCGGCAC-3′	L13266	60°C
	5′-ATGTCTGCCTGCCCGCTG-3′		
Tau	5′-ACCACAGCCACCTTCTCC-3′	NM_001123066	60°C
	5′-CAACCCGTACGTCCCAGC-3′		
TGF-α	5′-TCGCTCTGGGTATTGTGTTG-3′	NM_001099691	60°C
	5′-CCATGGAAGCAGAACTGAGT-3′		
FGF-4	5′-CCACCTCCAGGCGCTCCC-3′	NM_002007	60°C
	5′-TGCTCATGGCCACGAAGAAC-3'		
GAPDH	5′-CATCATCTCTGCCCCCTCT-3′	NM_002046	60°C
	5′-CAAAGTTGTCATGGATGACCT-3′		

### Western Blotting analysis

SDS-polyacrylamide gel electrophoresis (SDS-PAGE) was carried out according to Laemmli [38]. The NT2 cells were seeded and cultured, as spheres, on bacterial Petri dishes and treated for 5 weeks in 3 different conditions: they were grown in absence (control) and in presence of Ca^2+^-ICR frequency (exposed) and also treated with retinoic acid which was used as positive control. Equal amount of proteins from control, exposed and RA treated cells were loaded for each lane, after lysis in sample buffer (1505]mM NaCl, 505]mM Tris/HCl pH8, 0.55]mM EDTA, 0.15]mM EGTA, Triton X-100 1%). Electrophoresis was carried out in a range between 6-12% SDS polyacrilamide gel at 605]V for 2 hours. Transfer on nitrocellulose membranes (Biorad) was subsequently performed at 300 mA for 2 hours. After blocking in 5% fat-free-milk for 1 hour at room temperature, membranes were incubated with the following monoclonal antibodies: anti-NeuroD (1∶1000), anti-NR1 (1∶500), anti-Tau (1∶1000), anti-NF200 (1∶200) and anti-Cripto-1 (1∶100) and revealed by chemiluminescence (ECL) system (Amersham).

### Soft agar colony formation assay

Anchorage-independent growth was assessed with soft agar colony formation assay using the CytoSelect 96-Well Cell Transformation Assay Kit (Cell Biolabs) following the instructions of the manufacturer. The NT2 cells were seeded and cultured on bacterial Petri dishes as spheres and treated for 5 weeks in 3 different conditions: they were grown in absence (control) and in presence of Ca^2+^-ICR frequency (exposed) and also treated with retinoic acid which was used as positive control. The 5 week old cellular spheres were disaggregated and suspended in DMEM containing 0.4% low-melting agarose and 10% FBS and then seeded on a substrate composed of DMEM containing 1% of low-melting agarose and 10% FBS. Cultures were maintained for 7 days. Colony formation was measured by agar solubilization followed by cell lysis and quantification of cell number by use of CyQuant GR Dye in a fluorescence plate reader. Data were reported as fluorescence intensity, which is directly proportional to cell number.

### Statistical analysis

The SPSS (Statistical Package for the Social Sciences) software, version 20, was used for statistical analysis, and the significance level adopted for all analyses was P<0.05.

For the results of cell metabolic activity, cell proliferation, RT-PCR and Western Blot analysis, data were analyzed by two-way ANOVA test (Time×Treatment with time as a repeating variable) followed by one-way ANOVA test at each week point to verify the statistical significance among different groups (control, exposure, and RA treatment). For the week points which were statistical significant in the one-way ANOVA test a subsequent Tukey post hoc test (p<0.05) was used to verify significant comparisons between different treatment groups. For Ki67 mRNA expression analysis and soft agar assay, data were analyzed by one-way ANOVA test followed by the Tukey post hoc test.

## Results

### Effect of Ca^2+^-ICR frequency on NT2 cell Morphology

**Figure 2:**
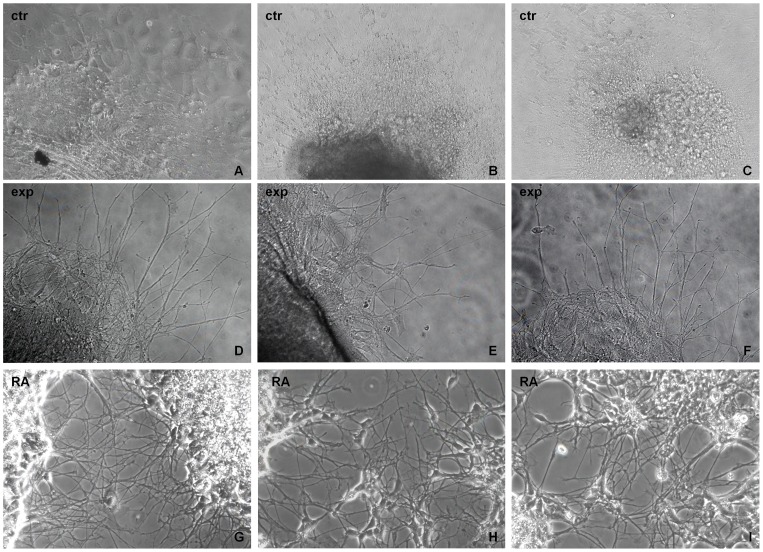
Effect of Ca^2+^-ICR exposure on NT2 cell morphology by phase contrast microscope analysis. (A)The control (ctr), (B) exposed (exp) and (C) retinoic acid (RA) treated NT2 cells were grown as spheres for 5 weeks and seeded on Petri dishes in a matrigel substrate to study their cell morphology. Exp NT2 cells similarly to the RA treated cells show a differentiated cellular morphology in which the cells have developed some neuritic like-structures (×20 objective).

The human NT2 cells were grown as spheres for 5 weeks in three different conditions (ctr, exp and RA), then seeded in a matrigel substrate on Petri dishes to study their cell morphology. In this condition the development of cellular floating aggregates into adherent spheres was obtained. Control cells appeared as an amassed central growth with the external monolayer having an undifferentiated morphology, without neuritic-like structures, (Figure 2A-C). Instead the NT2 cells exposed to Ca^2+^-ICR frequency showed a differentiated cellular morphology with the development of some neuritic like-structures beginning to organize a neuronal network. These structures were started off by the single cells which are present in the outer part of the adherent spheres (Figure 2D-F). In addition, similar structures having a well organized neuronal network were developed in our positive control, the NT2 cells treated with retinoic acid (Figure 2G-I).

### Effect of Ca^2+^-ICR frequency on NT2 cell metabolic activity and proliferation

**Figure 3:**
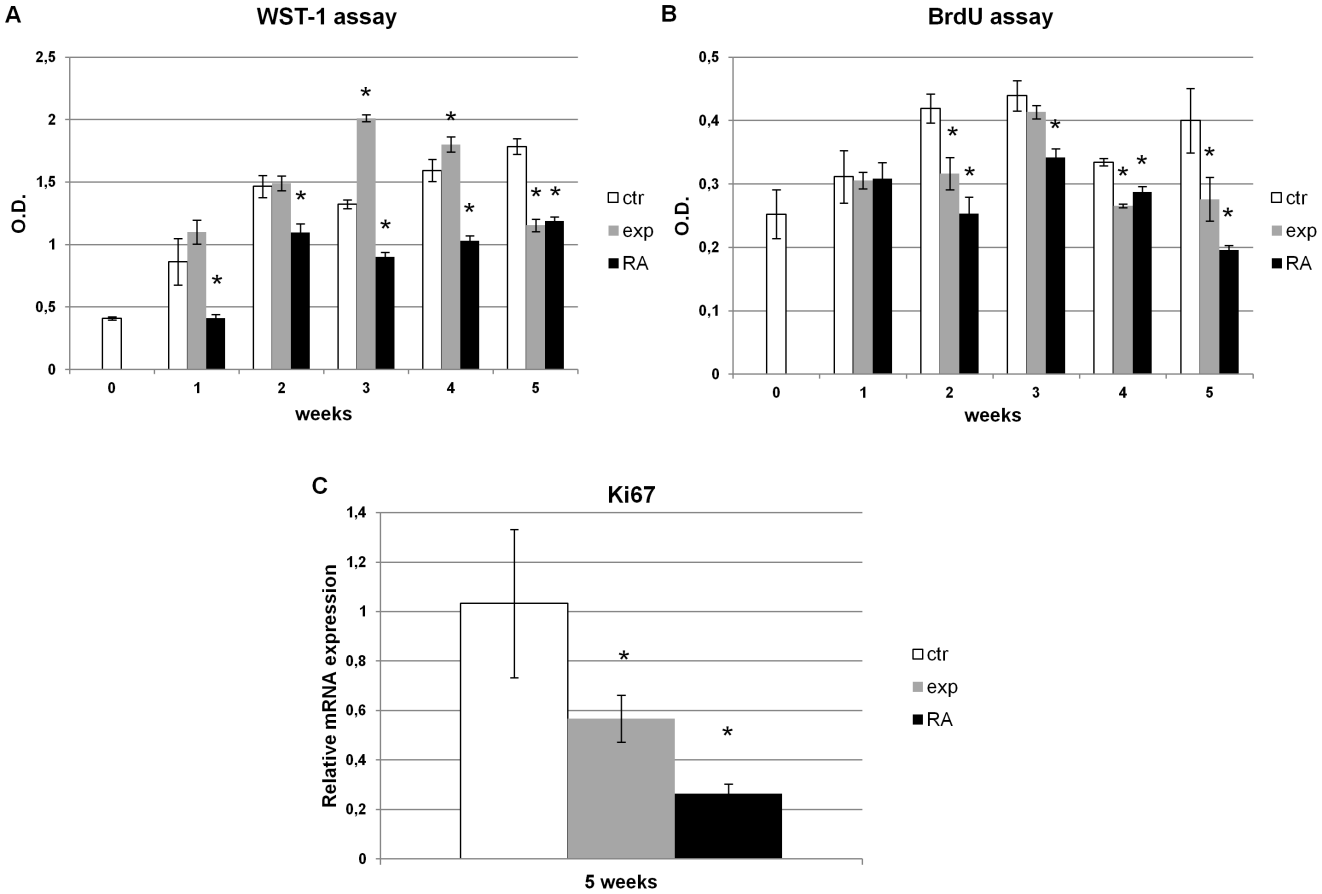
Effect of Ca^2+^-ICR frequency on NT2 cell metabolic activity and proliferation. (A-B) The Ca^2+^-ICR exposed cells increased their metabolic activity and the proliferation trend from week 1 to week 4 compared to the control ones followed by a statistical significant decrease at the 5^th^ week of treatment. The proliferative status of these cells at week 5 was also studied analyzing the expression of the Ki67. (C) Data are shown as mean ± standard deviation (SD). Asterisks identify statistical significance referring to the control sample (P<0.05).

Retinoic acid treated NT2 cells, grown for 5 weeks, showed a continuous decrease in metabolic activity compared to the control ones. The Ca^2+^-ICR exposed cells increased their metabolic activity trend from week 1 to week 4 compared to the control ones and then it decreased at the 5^th^ week of treatment as assessed by the WST assay (Figure 3A). To investigate the proliferative status of these cells we performed the bromodeoxyuridine incorporation assay and analyzed the expression of the Ki67, a well-characterized proliferation marker. BrdU-pulse experiments on NT2 exposed cells revealed a lower proliferation rate compared to control cells. This effect started to appear at week 2 and reached its peak at week 5 similarly to the RA treatment (Figure 3B). Furthermore, the RT-PCR analysis performed at week 5 for the proliferation marker Ki67 revealed that a lower metabolic activity and proliferation rate also correspond to a decrease of the Ki67 mRNA expression (Figure 3C).

### Effect of Ca^2+^-ICR frequency on mRNA neuronal differentiation marker expression in NT2 cells

The mRNA levels of early and late neuronal differentiation markers were studied by RT-PCR analysis on NT2 cells.

**Figure 4:**
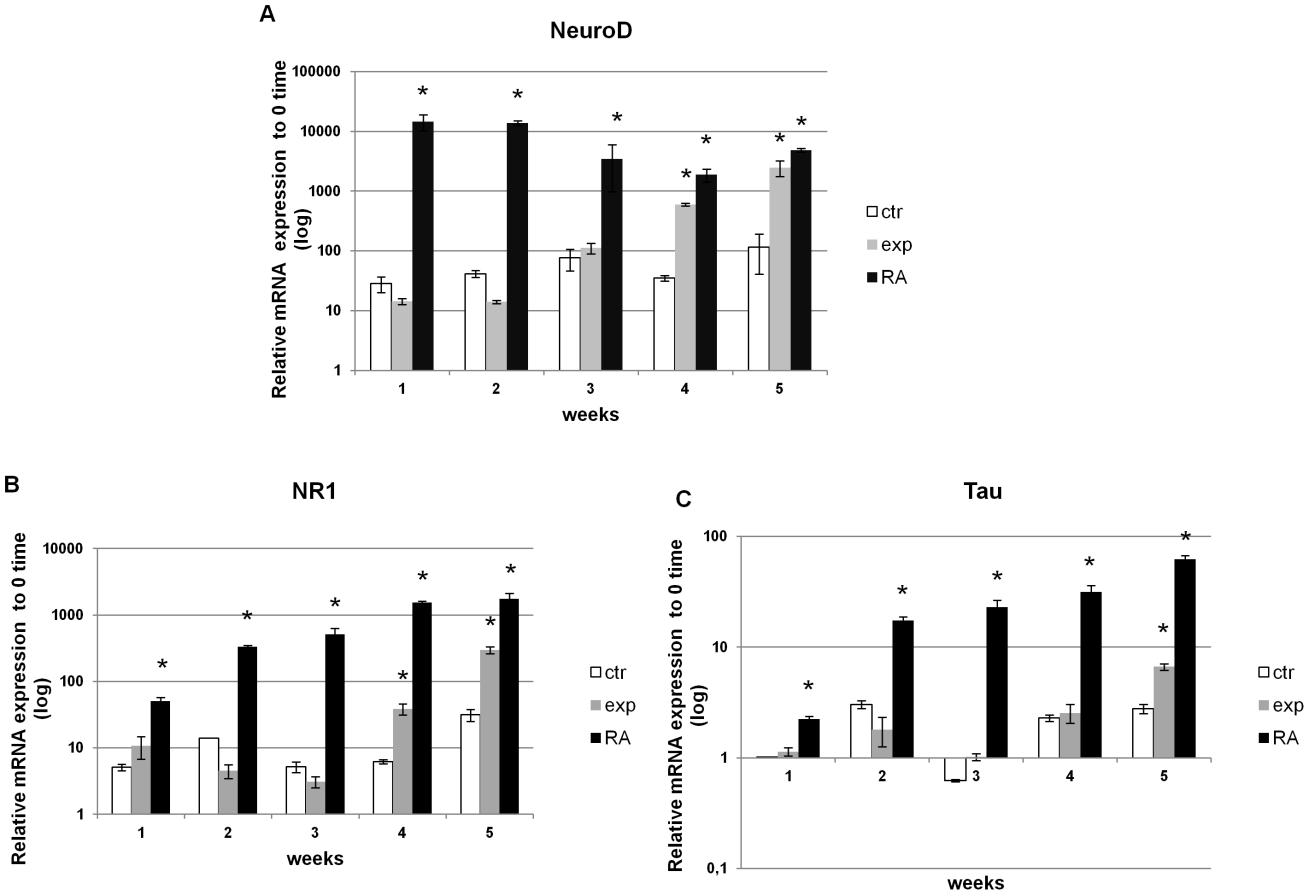
Effect of Ca^2+^-ICR exposure on NT2 cell mRNA expression of neuronal-differentiation markers. (A) Results of the Real Time-PCR analysis in three different conditions of NeuroD, NR1 and Tau mRNAs from 1 to 5 weeks of treatment (A-C). Data are shown as mean ± standard deviation (SD). Asterisks identify statistical significance referring to the control sample (P<0.05).

The RA differentiation treatment induced NeuroD, NR1 and Tau mRNAs to increase in NT2 cells during the 5 weeks of treatment. The Ca^2+^-ICR exposure acted on NT2 cells mostly at late time as demonstrated by a slower but constant increase of the NeuroD expression compared to the RA treatment (Figure 4A). Particularly the increase of the early neuronal differentiation marker NeuroD, in the exposed NT2 cells resulted statistically significant at week 4 and 5 compared to control cells.

The Ca^2+^-ICR exposure also induced the late neuronal differentiation marker expression NR1 and Tau to increase compared to control cells following the same but lower trend obtained by the RA treatment (Figures 4B and 4C).

### Effect of Ca^2+^-ICR frequency on neuronal protein expression in NT2 cells

**Figure 5:**
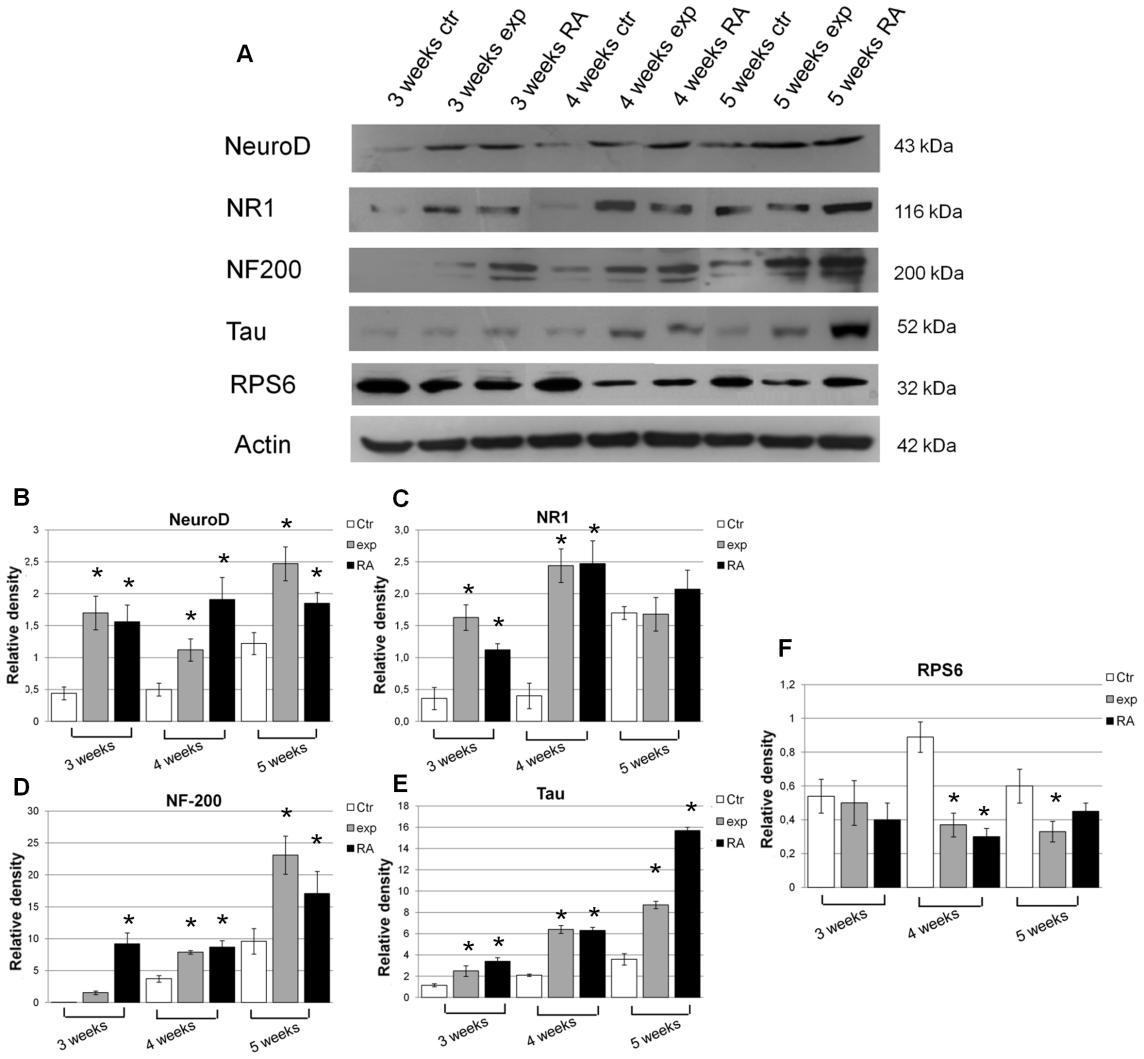
Effect of Ca^2+^-ICR exposure on NT2 cell protein expression of neuronal-differentiation markers. (A) Western Blot results performed in the three different conditions, from 3 to 5 weeks, using anti- NeuroD, Tau, NR1, RPS6 and NF-200 antibodies. (B-F) Semiquantitative analysis by VersaDoc using Quantity One software. Data are shown as mean ± standard deviation (SD). Asterisks identify statistical significance referring to the control sample (P<0.05).

The neuronal differentiation markers NeuroD, NR1, NF-200, Tau and RPS6 were also studied by western blot analysis (Figure 5A-F).

In Figure 5, the exposed cells, showed an increased levels of the NeuroD, NR1, NF-200 and Tau protein at 3, 4 and 5 weeks of exposure when compared to control ones. The same trend was also observed in the RA treated cells. The exposed cells also showed a down-regulation at week 3, 4 and 5 of the ribosomal protein S6 (RPS6) confirming and supporting the above mentioned results (Figure 5F).

### Effect of Ca^2+^-ICR frequency on Cripto-1 protein expression in NT2 cells

Cripto-1 protein expression levels were studied by western blot analysis on NT2 cells.

Control cells, showed a significant statistical increase in the Cripto-1 protein expression during the 5 weeks of culture.

**Figure 6:**
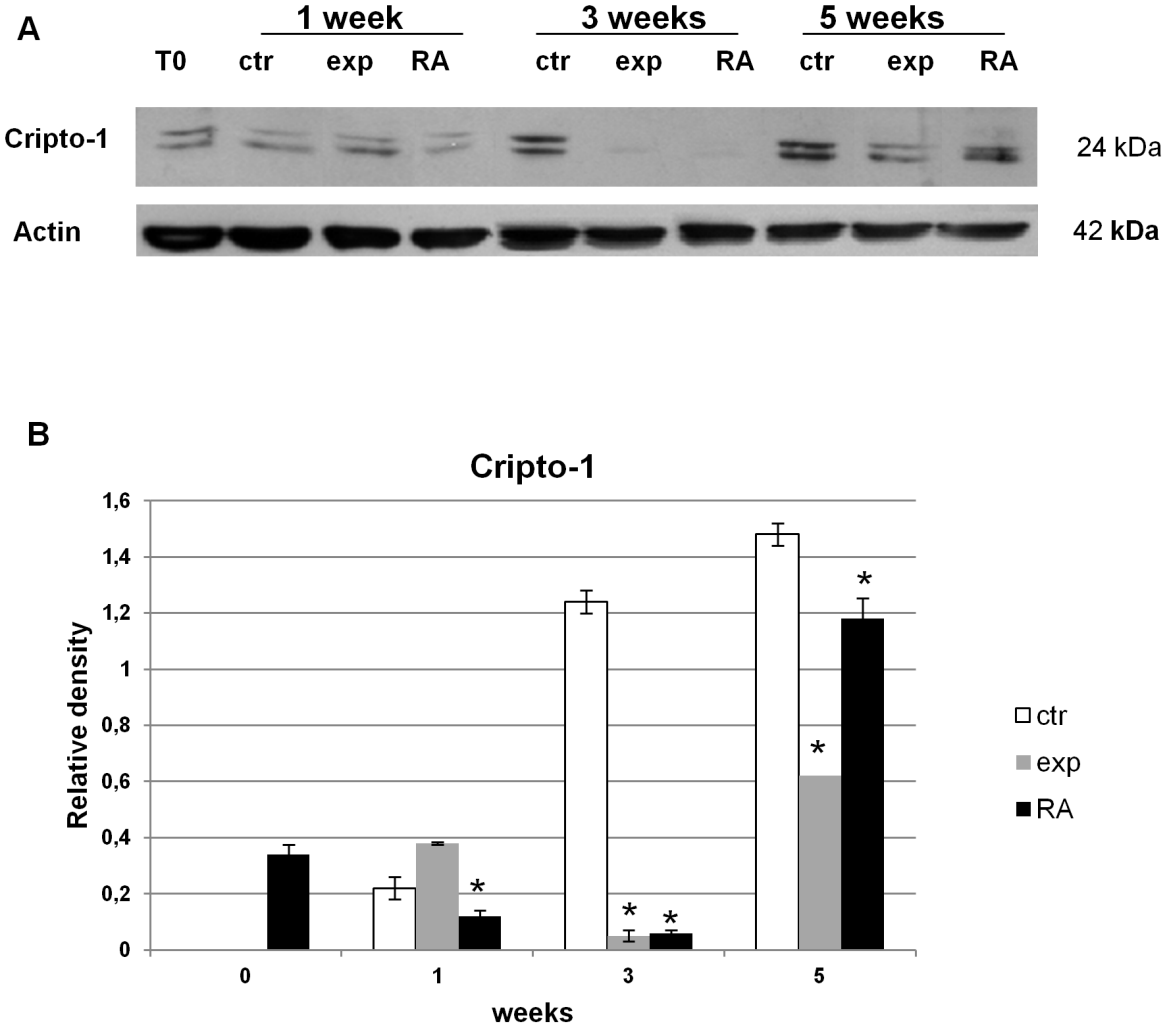
Effect of Ca^2+^-ICR exposure on NT2 cell Cripto-1 protein expression. (A) Western Blot results at 1, 3 and 5 weeks of treatment in three different conditions using anti-Cripto-1 antibody. (B) Semiquantitative analysis by VersaDoc using Quantity One software. Data are shown as mean ± standard deviation (SD). Asterisks identify statistical significance referring to the control sample (P<0.05).

Both retinoic acid and Ca^2+^-ICR exposure treatments induced a strong decrease of the Cripto-1 protein expression during the late phase of treatment compared to the control NT2 cells. Particularly, at week 3 it disappeared completely (Figure 6A-B).

### Effect of Ca^2+^-ICR frequency on mRNA expressions of Transforming and Fibroblast Growth Factors (TGF-α and FGF-4) in NT2 cells

**Figure 7:**
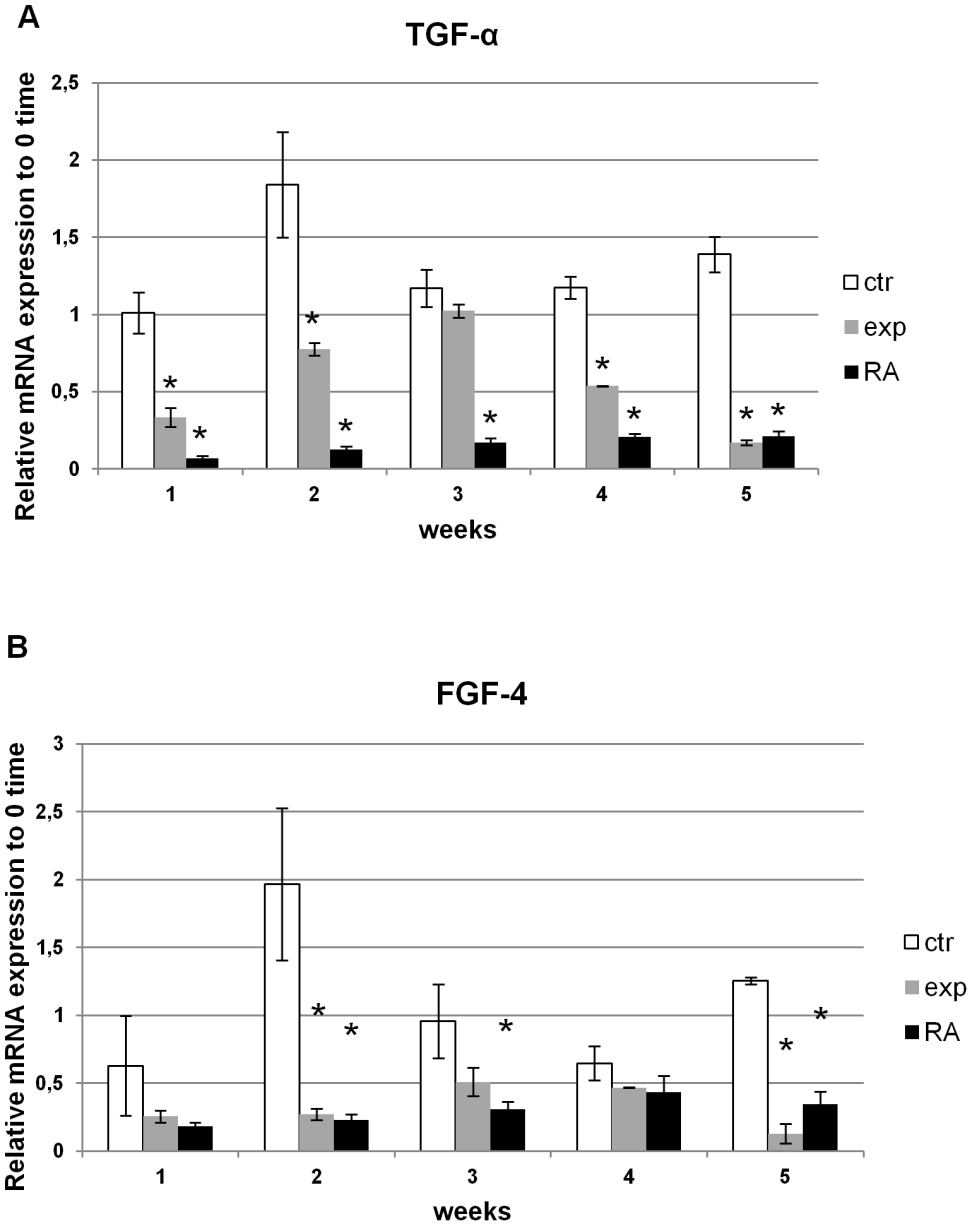
Real Time-PCR analysis for TGF-α and FGF-4 expression on Ca^2+^-ICR exposed NT2 cells. (A-B) TGF-α and FGF-4 mRNAs expression analysis of cells in three different conditions treated for 1 to 5 weeks. Data are shown as mean ± standard deviation (SD). Asterisks identify statistical significance referring to the control sample (P<0.05).

The mRNA levels of FGF-4 and TGF-α, were studied by RT-PCR on NT2 cells. Ca^2+^-ICR exposed cells from week 1 to 5 showed the same decreasing trend of the FGF-4 and TGF-α mRNA marker expressions found also in the NT2 cells treated with RA. This down-regulation was statistically significant and reached its peak in exposed cells at week 5 similarly, if not better, to the cells differentiated with the RA treatment (Figure 7A-B).

### Effect of Ca^2+^-ICR frequency on NT2 cell colony formation in soft agar

**Figure 8:**
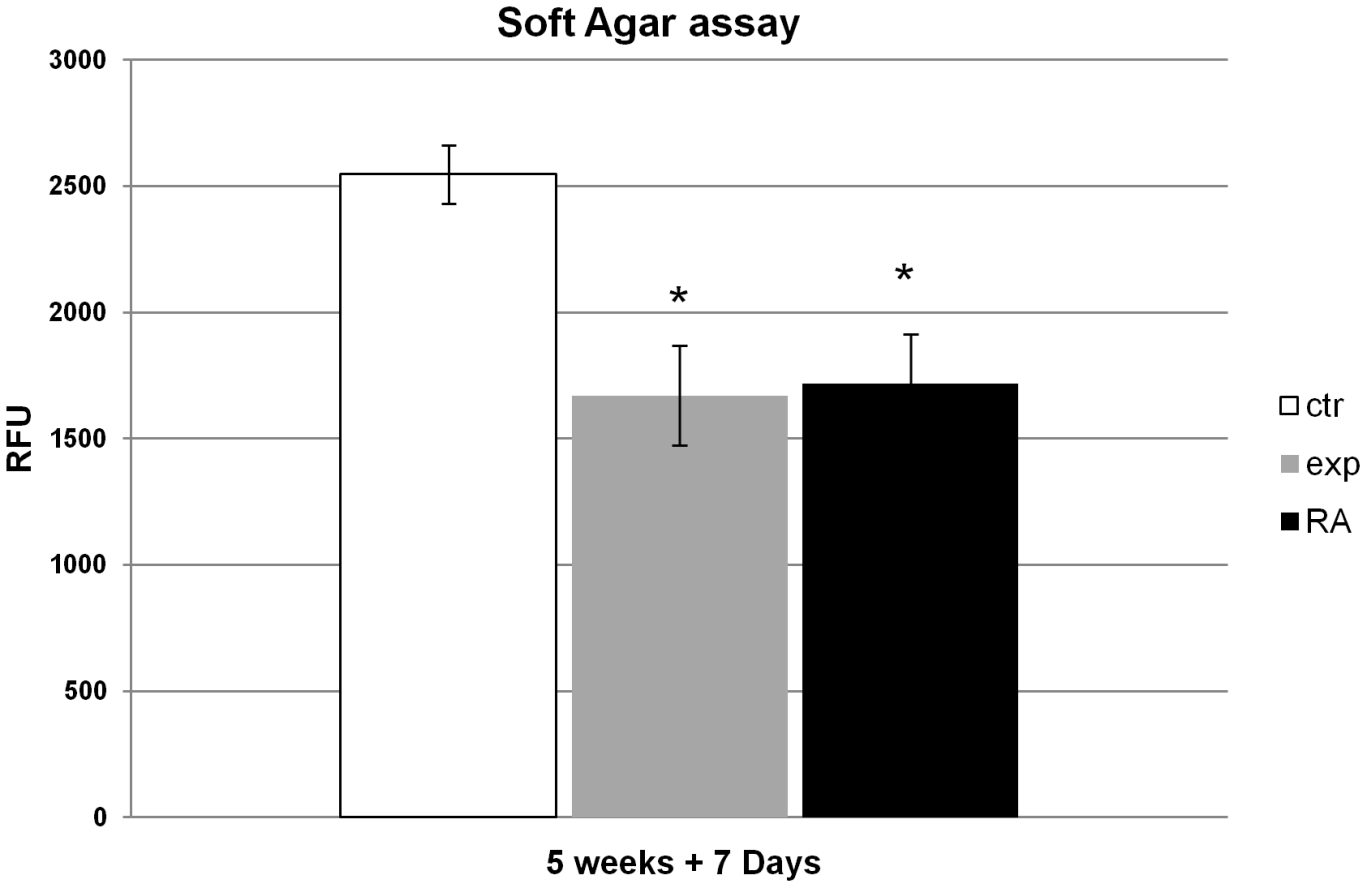
Effects of Ca^2+^-ICR exposure on NT2 cell colony formation in soft agar. The NT2 cells were grown as spheres for 5 weeks in three different conditions (ctr, exp, RA) and then seeded in soft agar substrate for another 7 days to study their capability of forming colonies which was measured using CyQuant GR Dye in a fluorescence plate reader. Data were reported as fluorescence intensity which is directly proportional to the cell number. Data are shown as mean ± standard deviation (SD). The asterisks identify statistical significance referring to the control sample (P<0.05).

The ability of NT2 exposed cells to form colonies in soft agar was evaluated. The cellular spheres were cultured and treated for 5 weeks in 3 different conditions: in absence (control), in presence of Ca^2+^-ICR frequency (exposed) and also treated with RA which was used as positive control. The 5 week old exposed NT2 cells, seeded and grown for 7 days in soft agar, showed a decrease in their capability to form colonies when compared to control ones, similarly to those treated with the retinoic acid (Figure 8).

## Discussion

In this paper we studied the effect of Ca^2+^-ICR frequency on NT2 exposed cells demonstrating an increase of the neuronal differentiation process and a reduction of their tumorigenicity.

More importantly, we performed this study using an exposure system inside a µ-metal shielded room as a magnetic field generator in order to remove the divergence in methods and results and to increase the reliability and the clinical feasibility of EMF-based technologies. Presently this knowledge has still not progressed into clinical applicability because of differences in experimental exposure protocols due to static MF variations producing dissimilar data which generates skepticism. Using our exposure system we were able to obtain totally controlled conditions and consistent results which enabled us to present these relevant data.

The biological effects of the electromagnetic field interaction on biological systems were widely studied and reported. Specifically the effects on cell proliferation [29], [30], intracellular calcium variation [32] and cell differentiation [31], [32], [33], [34] have been thoroughly investigated by us and by the above mentioned authors. The proliferation and differentiation processes are both related to changes in the intracellular Ca^2+^ influx and, as reported in our previous studies, we demonstrated that the exposure of pituitary corticotrope-derived cells to ELF-EMFs stimulates a statistically significant increase in intracellular calcium ([Ca^2+^]i) followed by a drop in intracellular pH (pHi) [39]. In these cells the ELF-EMFs are able to induce differentiation through the L-type voltage-gated Ca^2+^channels (VGCCs) which also play a pivotal role in C2C12 muscle cell differentiation [30] or, as reported by other authors, to directly affect the Ca^2+^ transporters or Ca^2+^ release from intracellular stores through phospholipase C [40]. The involved mechanism able to explain this effect is still unknown, but as it can be seen, it is strongly linked to the properties of the membrane channels [41] explained as Ca^2+^ flux alterations through the membrane which are due to the diamagnetic anisotropic properties of membrane phospholipids. Indeed Lednev reported that the reorientation of these molecules during the exposure to EMF, could result in the deformation of embedded ion channels and in the alteration of their dynamics. In particular he considers as a dipole, an ion in its protein-binding site; when the ion is exposed to its ICR frequency, the energy is transferred to the dipole and, as a consequence, the ion is released in the solution [42]

According to this hypothesis, we used Ca^2+^-ICR frequency as a physical differentiation factor to induce neuronal commitment in the NT2 cells grown as cellular floating aggregates. We demonstrated that NT2 cells change their morphology after exposure similarly to the RA treatment. These cells become bi-or multi-polar with smaller cell bodies and develop neuritic-like structures, the typical morphology of neuronal cells (Figure 2D-F). In addition, exposed cells, showed a higher metabolic activity and a higher cell proliferation trend at week 1, 2 and 3 (Figure 3A-B) followed by a lower trend from week 4 to 5 of exposure if compared to control cells. These results were also confirmed by the Ki67 mRNA expression that shows a decrease at week 5 (Figure 3D).

The proliferation and differentiation processes are linked together, as in the neuronal differentiation of NT2 cells, because the end of the proliferation process corresponds to the beginning of the differentiation process. Therefore, the effect on NT2 cell proliferation induced by the EMF, could be the basis of the beginning of their differentiation, later confirmed at transcriptional and translational level in this study (Figures 4 and Figure 5). As a matter of fact the early and late mRNAs of neuronal differentiation markers NeuroD, NR1 and Tau were up-regulated in exposed NT2 cells. In particular, their messenger expressions show a statistically significant increasing trend compared to the control ones which reach, in the majority of markers at week 4 and 5, the same expression level of the RA treatment. The neuronal differentiation process induced by the Ca^2+^-ICR exposure in these cells was supported also at protein level confirming the increase of NeuroD, NR1, Tau and NF-200 proteins. In our study, the neuronal commitment, was also verified through the modulation of the ribosomal protein S6 (RPS6) expression. Indeed, as reported by Bevort et al [43], the decrease of the mRNA expression of ribosomal protein S6 is related to either the decrease of proliferation or the increase of differentiation and it is down-regulated during the RA-induced NT2 cell differentiation.

We highlighted a statistically significant decrease of the S6 expression in the last two weeks of exposure when the NT2 cells showed a significant increase of neuronal differentiation markers (Figure 5A, F).

The NT2 post-mitotic differentiated cells, are a safe alternative to embryonic tissue [12], [44], [45] and have clinical application in tissue repair and regeneration as demonstrated by preliminary studies for transplantation therapy in strokes [46], [47].

It is a complex process to differentiate these cells to neuron-like cells from a fast growing carcinogenic cell line. Studies have demonstrated that undifferentiated NT2 cells, after RA treatment decrease their proliferation rate with a decrease of both TGF-α and FGF-4 along with a reduction in both tumor formation and the generation of new tumors in a-timic mice [10], [16], [17], [48]. Accordingly we found that the Ca^2+^-ICR exposure is able to induce a down-regulation of the TGF-α and FGF-4 messenger expressions at week 5 compared to untreated cells (Figure 7A-B) highlighting, during the first 3 weeks of exposure, a higher expression, that then decreases to the same RA expression level at weeks 4 and 5 (Figure 7A).

The human Cripto-1 gene, overexpressed in some types of cancer [49], [50], [51], functioning as a transforming gene [52], is also expressed in growing NT2 undifferentiated cells where it acts as a potent oncogene. In these cells, the RA-induced differentiation results in the loss of malignant growth with the repression of tumorigenicity and this occurs simultaneously with the down-regulation of TGF-α, FGF-4 and Cripto-1 expressions [16], [17]. In the present study, we also evaluated the effect of Ca^2+^-ICR-induced NT2 differentiation on the Cripto-1 gene expression. We found that (Figure 6A-B) this physical agent is able to down-regulate the Cripto-1 expression efficiently, as in the RA treatment, but for a longer time. Moreover, the repression of tumorigenicity of the exposed cells was also valuated by their capability of developing colonies in soft agar which show a reduction in their developing capability confirming our results (Figure 8).

In conclusion, we demonstrated that Ca^2+^-ICR exposure modulates cell proliferation, neuronal differentiation and tumorigenicity of NT2 cells. Even if further studies are required to fully understand this effect, our findings open up the potential therapeutic importance of the Ca^2+^-ICR exposure in regenerative medicine and in the germ tumor treatment of patients who do not respond to the current available therapeutic strategies.
